# Never bored!

**DOI:** 10.1117/1.NPh.11.3.030101

**Published:** 2024-09-30

**Authors:** Anna Devor

## Abstract

Neurophotonics Editor-in-Chief Anna Devor reflects on the wonderful feeling of inspiration in the neurophotonic community.

I like sweet bell peppers. I like them raw and whole, like apples. So, here I am on Sunday night at Trader Joe’s checkout line admiring colorful bell peppers on top of a few other items in my basket. The line moves quickly, and before I know it a friendly looking Crew with a pierced eyebrow waves me to the register.

“Beautiful day today,” he says taking the bell peppers out of my basket.

“Yes, lovely day. I’m sorry you are stuck indoors,” I say. “I was also indoors all day,” I add.

“Well, I’m reminding myself that I chose to be here,” he says. “What do you do?” he asks with a look at the leather messenger bag over my shoulder.

“I’m a professor at BU.” I keep my voice down but a very young-looking Crew at the next register turns around. It’s late, the store is about to close, and there are no more shoppers in line. I give a shrug of resignation. “I was working on a paper,” I say apologetically.

The pierced Crew places my items into a tote and looks at me like an anthropologist who just came across a peculiar human specimen.

“What do you study?” asks the young Crew.

“Brain. I study the brain. We call it ‘neurophotonics.’ It’s *neuro*-engineering.”

“You must be in high demand,” says the pierced Crew and gives me the tote. “You probably are never bored.”

“No.” I smile and shake my head vigorously. “Never bored.”

“So, how would you describe yourself in one word?” the young Crew says looking intensely into my eyes.

This small talk has taken a deep dive. What is the right word? Oh, that’s too easy. “Inspired!” I say.

Now both Crew smile. I put the tote over my shoulder and my new friends wave me goodbye. The doors of Trader Joe’s are locking behind me.

I walk to my car. Yes, the word that came to my mind describes exactly how I feel about science. It also describes how I feel about the team I’m doing the science with. Our team just had our annual retreat, which we call Blood Fest, with trainees and guests. We had great time. There is so much talent in our team and the neurophotonic community as a whole! I feel that we, the community, have unprecedented tools at our disposal and are standing at the threshold of great things to come. This is a wonderful feeling.

**Figure:**
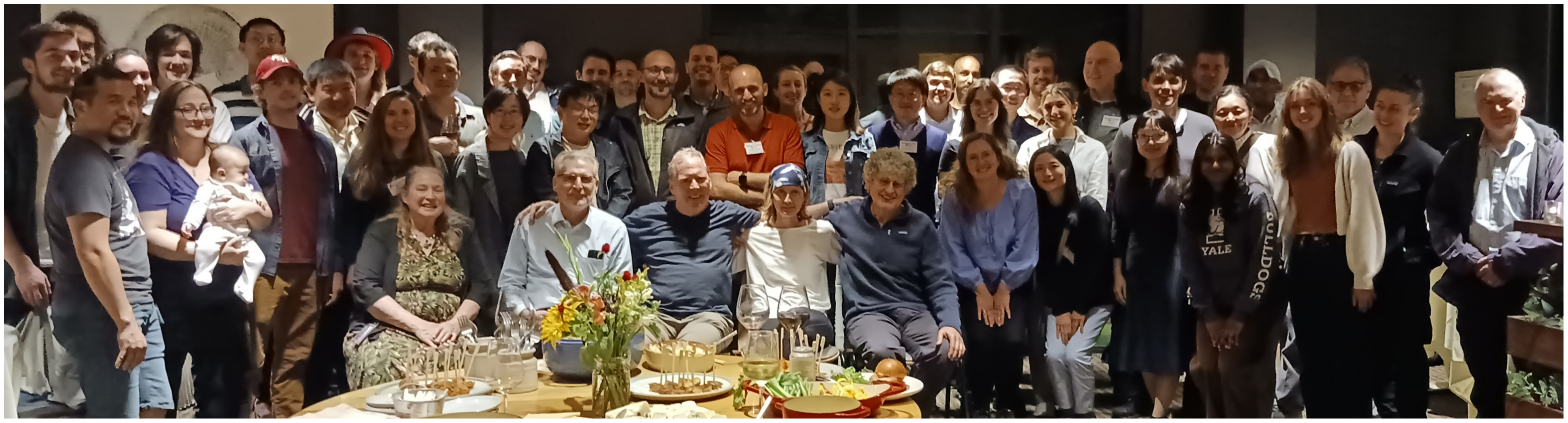
Team UltraSlow Arterial rhythms (USArhythms) and guests at Blood Fest 2024 in Burlington, Vermont.

The tote is on the back seat of my car. The bell peppers -- yellow, red and orange -- crown a pale pile of yogurts. Tomorrow is a new day with surprises, challenges, and mysteries to solve. No, we won’t be bored!

